# The complete chloroplast genome of *Hemisteptia lyrata* (Bunge) Fisch. & C. A. Mey. 1836 (Asteraceae) and its phylogenetic analysis

**DOI:** 10.1080/23802359.2024.2444624

**Published:** 2024-12-23

**Authors:** Chun-yan Li, Yan-ping Xing, Wen-juan Hou, Yu-tong Huang, Yan-chang Huang, Yue-yue Song, Ting-guo Kang, Yan-yun Yang, Liang Xu

**Affiliations:** School of Pharmacy, Liaoning University of Traditional Chinese Medicine, Dalian, China

**Keywords:** *Hemisteptia lyrata*, *Hemisteptia*, complete chloroplast genome, phylogenetic tree

## Abstract

*Hemisteptia lyrata*, widely distributed in China, has a 152,467 bp chloroplast genome with a large single-copy (LSC) region of 83,473 bp, a small single-copy (SSC) region of 18,594 bp, a pair of inverted repeat regions (IRs) of 25,194 bp in length. The GC content is 36.46%. A total of 133 genes were encoded, of which 88 were protein coding genes, eight were rRNA genes, and 37 were tRNAs. The phylogenetic analysis using the maximum-likelihood method showed that *H. lyrata* is closely related to the species in *Saussurea.* This study provides genomic resources for phylogenetic genetics and resource exploitation of *H. lyrata*.

## Introduction

*Hemisteptia lyrata* (Bunge) Fisch. & C. A. Mey. (1836) is an annual herb in Asteraceae, which was originally classified as *Saussurea*, but after a revision of the classification system was reclassified as *Hemisteptia*, with the name changed from *Saussurea lyrata* to *H. lyrata* (Shi and von Raab-Straube 2011). The whole herb of *H. lyrata* is used medicinally (Editorial Committee of the Chinese Materia Medica of the State Administration of Traditional Chinese Medicine 1998), with its main chemical constituents including flavonoids (Dong et al. 2012), lignans (Zou et al. 2006), among others. Studies have shown that flavonoids have antioxidant and free radical scavenging effects (Feng and Wang 2021), some studies have linked excess reactive free radicals to the development of diseases such as cancer and atherosclerosis (Tu et al. 2022). *H. lyrata* can inhibit lipid peroxidation induced by 80% chloroform–alcohol solution in mouse liver, with good antioxidant activities (Liao et al. 1999). *H. lyrata* is abundant in resources within our country and contains various bioactive compounds with promising clinical applications. This study is the first to molecularly analyze the complete chloroplast genome of mud horehound, confirming its phylogenetic position and offering valuable insights into its evolutionary relationships.

The chloroplast gene is circular DNA composed of four segments, IRA, IRB, LSC, and SSC, which provide information affecting chloroplast function (Liu et al. 2018). Chloroplast gene is characterized by high stability and independent inheritance, which can provide reliable basis for species identification and phylogenetic analysis (Lilly et al. 2001; Liu et al. 2023). Although the chloroplast gene of Asteraceae has been studied more and more, the whole chloroplast genome of *Hemisteptia* has not been studied. Studies on *H. lyrata* mainly focus on chemical composition and pharmacology, and related molecular research is only limited to some chloroplast genes, such as *matK* (Kita et al. 2004), and the whole chloroplast genome research is still blank. Therefore, in this study, the whole genome information of *H. lyrata* chloroplast was determined by next-generation sequencing technology, and the sequence characteristics, gene composition and phylogenetic relationships of this species were analyzed by bioinformatics software. This study laid a theoretical foundation for the study of genetic structure and genetic diversity of *H. lyrata*, and also provided support for future phylogenetic analysis and genetic diversity research of *Hemisteptia*.

## Materials and methods

**Figure 1.**
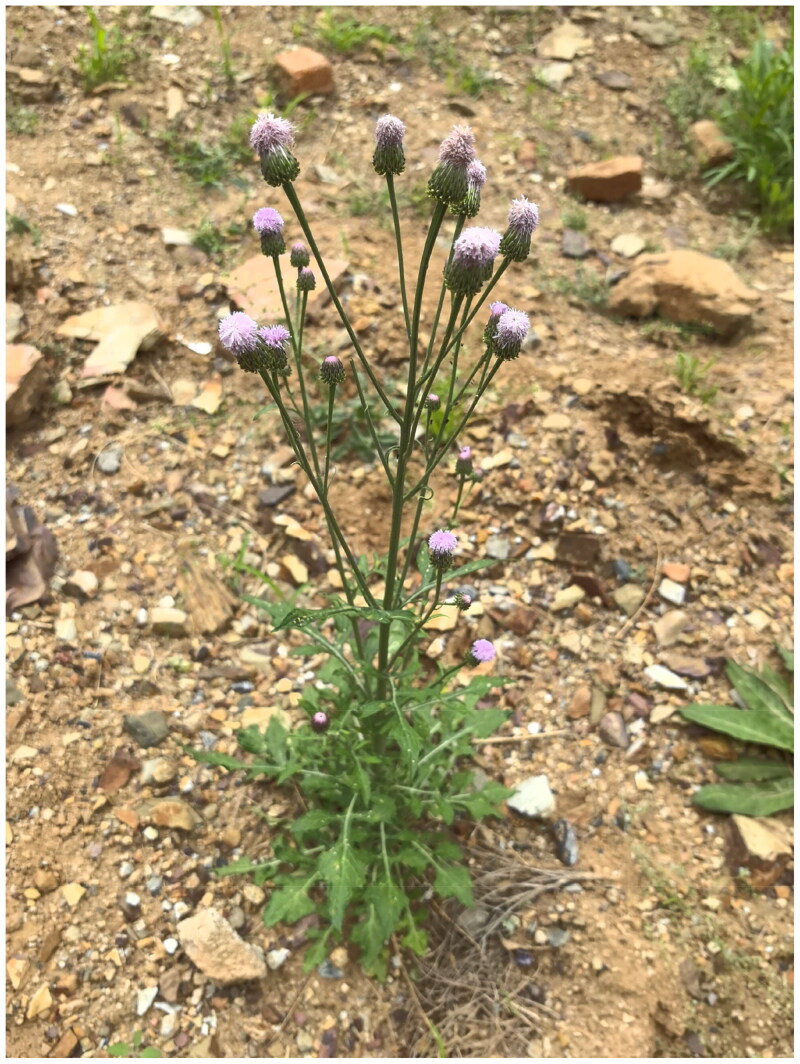
The morphological characteristics of *H. lyrata* were captured in a photograph by Chun-yan Li in Dalian, Liaoning, China (113°21′29.40″ E, 23°9′20.11″ N). Notable features of the specimen include large, pinnatipartite, or nearly divided leaves that are heterochromatic: green and glabrous on the upper surface, and grayish-white below. The florets are purple or reddish, with linear corolla lobes and a slender, tubular filiform section.

The fresh leaves of *H. lyrata* were collected in the Dalian region, China (113°21′29.40″ E, 23°9′20.11″ N) (Figure 1). Professor Tingguo Kang from the Liaoning University of Traditional Chinese Medicine, authenticated the sample vouchers. The specimen was deposited at the herbarium of Liaoning University of Traditional Chinese Medicine (Liang Xu 861364054@qq.com, *H. lyrata* number: 10162220524002LY) (Supplementary Figure S1). All operations were carried out in accordance with Specification on Good Agriculture and Collection Practices for Medicinal Plants (GACP; Number: T/CCCMHPIE 2.1-2018).

Total genomic DNA was isolated from fresh leaves utilizing the cetyltrimethylammonium bromide technique (Doyle and Doyle 1987). A total amount of 0.2 μg DNA per sample was used as input material for the DNA library preparations. Sequencing library was generated using NEB Next^®^ Ultra™ DNA Library Prep Kit for Illumina (NEB, Ipswich, MA) following manufacturer’s recommendations and index codes were added to each sample. Genomic DNA sample was fragmented by sonication to a size of 350 bp, and ligated with the full-length adapter for Illumina sequencing, followed by further PCR amplification. After PCR products were purified by AMPure XP system (Beckman Coulter, Beverly, MA), DNA concentration was measured by Qubit^®^ 3.0 Fluorometer (Invitrogen, Waltham, MA). The clustering of the index-coded samples was performed on a cBot Cluster Generation System using Illumina PE Cluster Kit (Illumina, San Diego, CA) according to the manufacturer’s instructions. High-quality reads were assembled into chloroplast genome using a de novo assembler SPAdes v3.14.1 (Bankevich et al. 2012).

The raw data were processed for quality control using the NGS QC Tool Kit v2.3.3 (https://nipgr.ac.in/ngsqctoolkit.html) (Patel and Jain 2012). High-quality sequence data were then selected, and the SPAdes assembler v.3.14.1 (http://cab.spbu.ru/software/spades/) was utilized from the start to assemble the complete chloroplast genome (Bankevich et al. 2012). Finally, it was annotated by PGA (Qu et al. 2019) with *Saussurea phaeantha* (MT554930) as reference genome.

The Illumina short sequences were compared to the chloroplast genome sequences using BWA software and finally the coverage was calculated using samtools depth (high coverage of over ×100). The horizontal coordinate is the chloroplast length and the vertical coordinate is the coverage depth (Supplementary Figure S2) (Li et al. 2009; Li 2013). The chloroplast genome maps of *H. lyrata*, including those of cis-splicing and trans-splicing genes, were generated using CPGview (Liu et al. 2023).

To analyze the relationship between *H. lyrata* and other species in the family Asteraceae, the whole chloroplast genomes of 29 species and two outgroup taxons (*Platycodon grandiflorus* and *Adenophora stricta*) were selected from NCBI. Using mafft-7.037 software (Katoh and Standley 2013), 70 shared protein genes were selected for comparison (Table S1). After sequence alignment using MAFFT, the sequences were then aligned using Gblocks 0.91b (Xiao et al. 2021), which is used to extract conserved sites from multiple sequence alignment results for the next evolutionary analysis. The maximum-likelihood evolutionary tree was then constructed using the iqtree-1.6.12 software (Xue et al. 2022) with a self-expansion value of 1000. The selection of the optimal substitution model (TVM + F + I + G4) was performed according to Bayesian information criterion (BIC) method implemented in ModelFinder (Kalyaanamoorthy et al. 2017).

## Results

**Figure 2.**
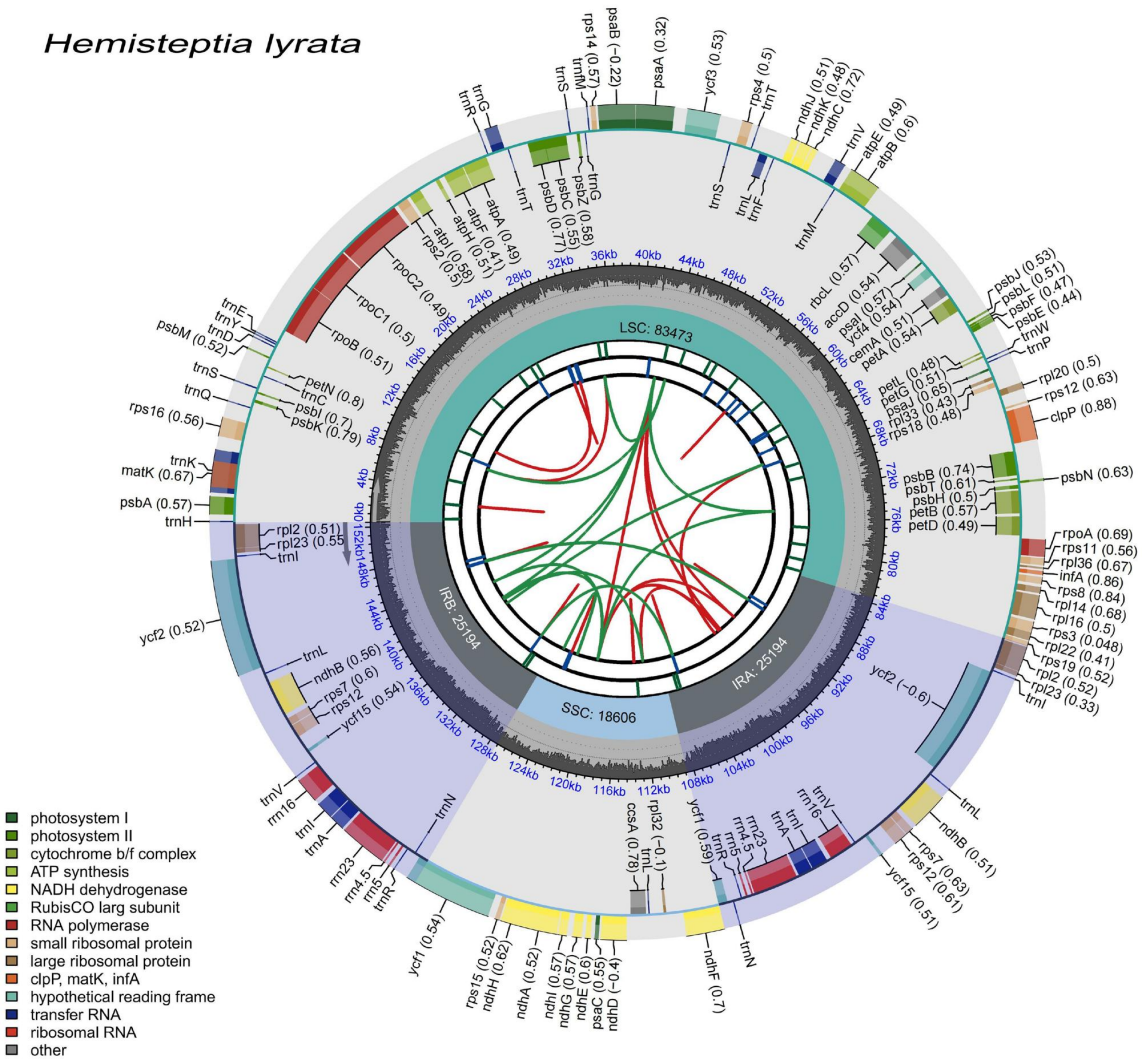
A schematic representation of the *H. lyrata* chloroplast genome. The graph is represented from inside out: (1) the green circle: LSC, the blue circle: SSC, the light grey circle: IRA and IRB; (2) the distribution of GC content on the chromosome; (3) the scale coordinate axis; (4) genes located on the negative strand and positive strands. And the numbers after the gene names indicate GC content of each gene.

After sequencing, we obtained a total of 4.59 GB of raw data, and with 96.53% of them having a Q20 quality score or higher, 4.56 GB of clean data were obtained after screening. The assembled complete chloroplast genome of *H. lyrata* was 152,467 bp in length, had an overall GC content of 36.46%, and average sequencing depth of 2242× (Figure S2). It exhibits a typical quadripartite structure, consisting of an 83,473 bp large single-copy (LSC), an 18,594 bp small single-copy (SSC), and 25,194 bp inverted repeats (IRs). The genome includes 133 genes, comprising 88 protein-coding genes, eight rRNA genes, and 37 tRNA genes. Sixteen genes (*trnK-UUU*, rps16, *rpoC1*, *atpF*, *trnG-UCC*, *rpoC1*, *trnL-UAA*, *trnV-UAC*, *petB*, *petD*, *rpl16*, *rpl2*, *ndhB*, *trnI-GAU*, *trnA-UGC*, and *ndhA*) contained one intron, three genes (*clpP* and *ycf3*) contained two introns, and rps12 gene was present in a trans-splicing situation. The *H. lyrata* chloroplast genome and an annotated map of cis- and trans-splicing genes (Figure 2, Supplementary Figures S3 and S4, respectively) were processed with CPGview (Liu et al. 2023). The chloroplast genome map clearly presents detailed information, including repeats, gene names, LSC, SSC, and IR regions. For instance, dispersed repeats are categorized into direct (D) and palindromic (P) repeats, which are represented by red and green arcs, respectively. The rest of the information is shown in Figure 2.

**Figure 3.**
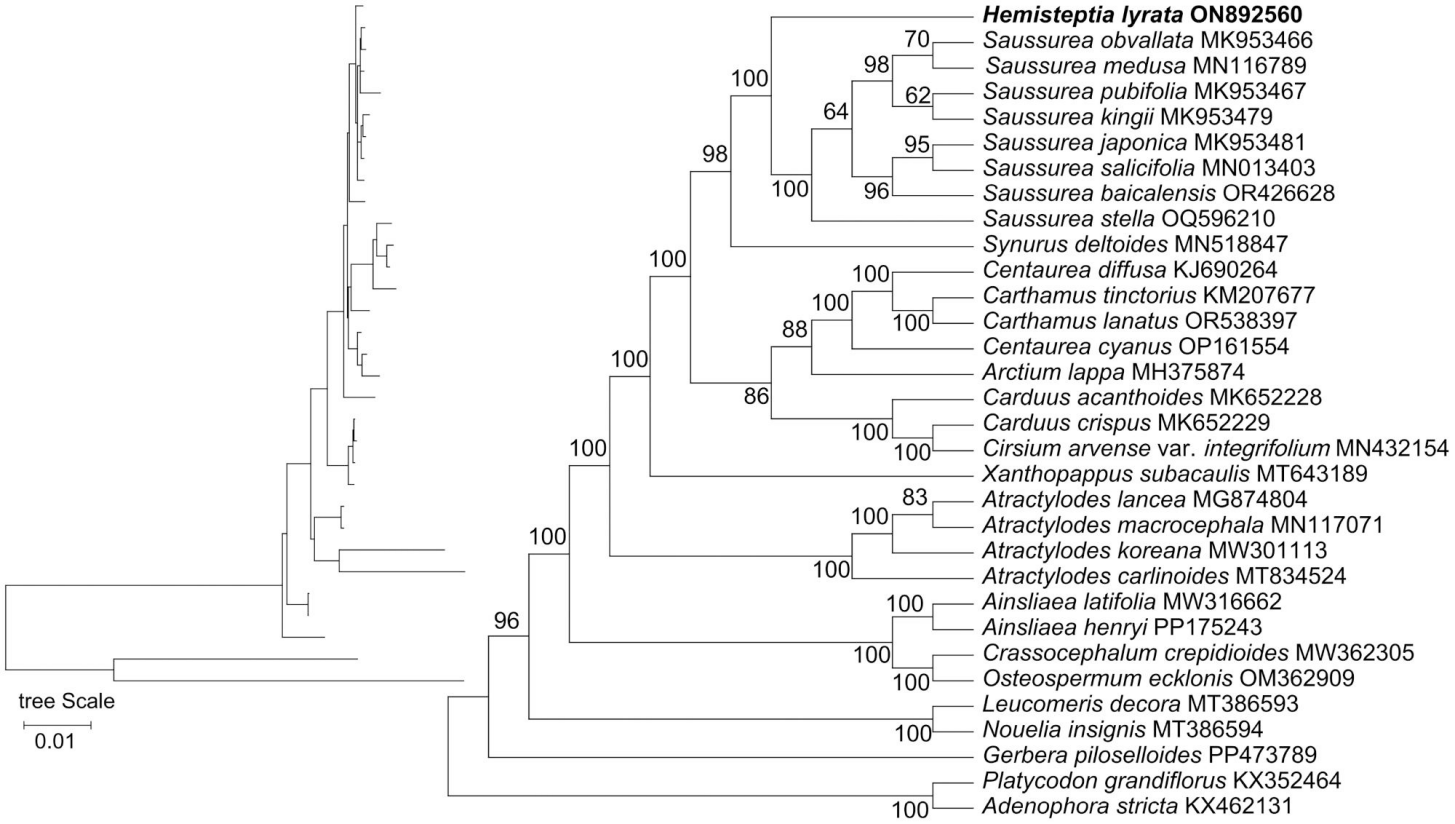
Maximum-likelihood (ML) phylogenetic tree with evolutionary distances for *H. lyrata* and 31 other species. The numbers above the branches show the bootstrap support values. The following sequences were used: *Arctium lappa* (MH375874), *Carduus acanthoides* (NC053725), *Carduus crispus* (NC053726), *Cirsium arvense* var. *integrifolium* (NC052868) (Xie et al. 2019), *Saussurea obvallata* (NC044726) (Zhang et al. 2019), *Saussurea pubifolia* (NC044727) (Zhang et al. 2019), *Saussurea stella* (NC072930), *Saussurea kingii* (NC044736) (Zhang et al. 2019), *Saussurea medusa* (NC052846), *Saussurea japonica* (NC044738) (Zhang et al. 2019), *Saussurea baicalensis* (NC083145), *Saussurea salicifolia* (NC057621) (Chen et al. 2019), *Xanthopappus subacaulis* (NC063322), *Carthamus lanatus* (NC085736), *Carthamus tinctorius* (NC030783), *Centaurea diffusa* (NC024286), *Centaurea cyanus* (NC066898), *Synurus deltoides* (NC046830), *Atractylodes lancea* (NC037483), *Atractylodes koreana* (NC056987), *Atractylodes carlinoides* (NC057124) (Wang et al. 2021), *Atractylodes macrocephala* (NC044671), *Osteospermum ecklonis* (NC061392), *Ainsliaea henryi* (NC086780), *Ainsliaea latifolia* (NC056135), *Gerbera piloselloides* (NC088558), *Leucomeris decora* (NC057205), *Nouelia insignis* (NC057206), *Crassocephalum crepidioides* (NC057993), *Platycodon grandiflorus* (NC035624), and *Adenophora stricta* (NC036223).

The topology of the phylogenetic tree revealed the affinities of *H. lyrata* with the selected 31 species. Phylogenetic tree results showed that *H. lyrata* was most closely related to the eight selected *Saussurea* plants. The establishment of the phylogenetic tree in this study lays the foundation for subsequent studies on Asteraceae (Figure 3).

## Discussion and conclusions

In this study, we present the complete chloroplast genome of *H. lyrata*, which consists of a quadripartite structure totaling 152,467 bp. The basic structure of conserved chloroplast genes is consistent with the results of previous studies on chloroplast genes in vascular plants (Palmer and Stein 1986; Daniell et al. 2016). Phylogenetic analysis shows that *H. lyrata* is closely related to eight species of *Saussurea*, with the clustering pattern of the Asteraceae family consistent with previous studies (Park et al. 2019). The phylogenetic tree shows that the plant is closely related to the *Saussurea*, which accords with the traditional taxonomy. In traditional taxonomy, *H. lyrata* and *Saussurea* both belong to the Saussurea group and exhibit substantial morphological similarities. These include alternate leaf arrangement, capitulum inflorescence, bisexual flowers, and the presence of hair-like structures on the receptacle. Both genera also share a multi-layered involucral bract structure and a two-layered, heteromorphic pappus. Despite these resemblances, there are notable differences in certain traits, such as the feathery outer pappus of *H. lyrata*, in contrast to the extremely short and rough outer pappus of *Saussurea* species (Shi and von Raab-Straube 2011). It is likely that the close morphological similarities and distinct differences in a few key traits initially led early taxonomists to classify *H. lyrata* under the *Saussurea*, before it was later transferred to the *Hemisteptia*. In addition, we found that the whole chloroplast genome of *Hemisteptia* is rarely reported, and further studies are necessary in the future.

The results of this study support traditional morphological classifications and provide valuable data for exploring evolutionary relationships within Asteraceae, as well as aiding in the development of *H. lyrata* germplasm resources. In the future, the chloroplast genome data of *H. lyrata* can be further utilized in phylogenetic studies and species identification. Additionally, chloroplast genes from *H. lyrata* may be applied in genetic engineering, potentially leading to the discovery of new medicinal properties.

## Supplementary Material

Supplementary materials.docx

## Data Availability

The genome sequence data that support the findings of this study are openly available in GenBank of NCBI at https://www.ncbi.nlm.nih.gov/ under accession no. ON892560. The associated BioProject, SRA, and Bio-Sample numbers are PRJNA854342, SRR19913670 (Illumina), and SAMN29432125, respectively.
