# Supplementary material for: The complete chloroplast genome of Hemisteptia lyrata (Bunge) Fisch. & C. A. Mey. 1836 (Asteraceae) and its phylogenetic analysis
Source: Mitochondrial DNA B Resour. 2024 Dec 23;10(1):6–10. doi: 10.1080/23802359.2024.2444624 (PMC11703484; doi:10.1080/23802359.2024.2444624)
Supplement: Supplementary materials.docx [file TMDN_A_2444624_SM2943.docx]

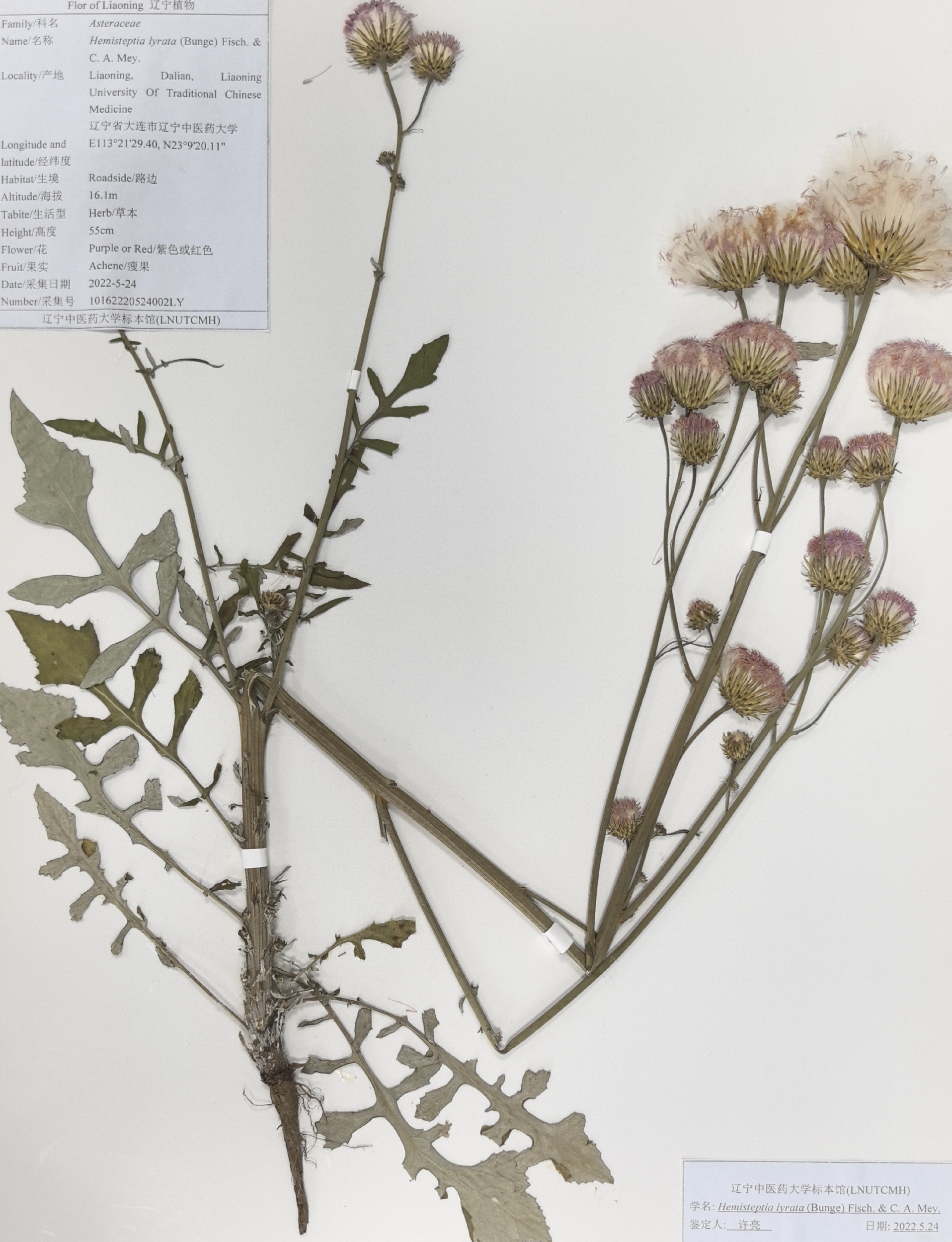


**Figure S1.** An illustration of *Hemisteptia lyrata* specimen preserved at Liaoning University of Traditional Chinese Medicine. Specimen number, collector, latitude and longitude and other details are noted on the map for reference.


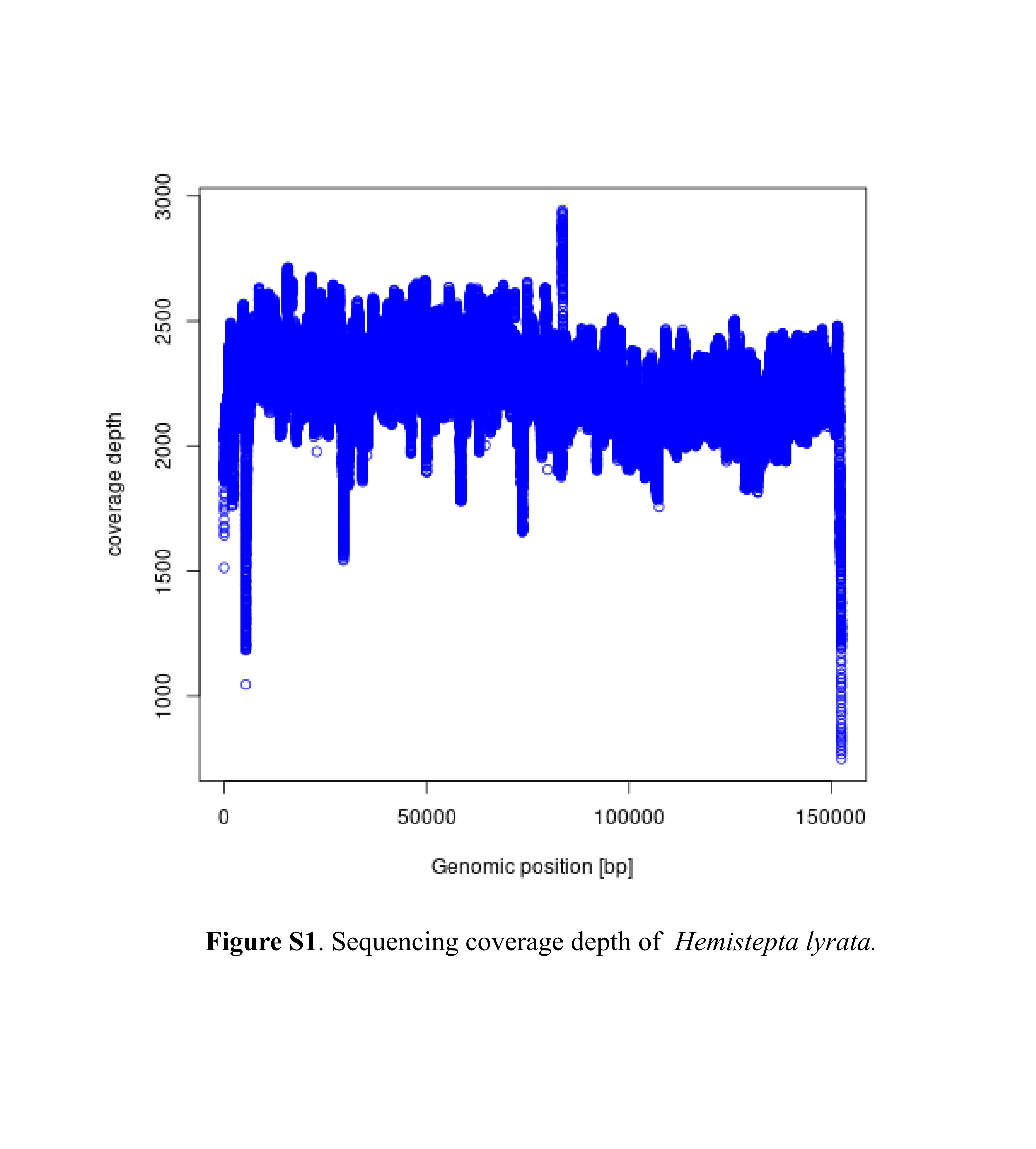


**Figure S2.** Sequencing coverage depth of *Hemisteptia lyrata.* The Illumina short sequences were compared to the chloroplast genome sequences using BWA software and finally the coverage was calculated using samtools depth (high coverage of over ×100). The horizontal coordinate is the chloroplast length and the vertical coordinate is the coverage depth.

Li H. 2013. Aligning sequence reads, clone sequences and assembly contigs with BWA-MEM. arXiv Prepr arXiv. 0(0):3.

Li H. et al. 2009. The Sequence Alignment/Map format and SAMtools. Bioinformatics, 25, 2078-2079.


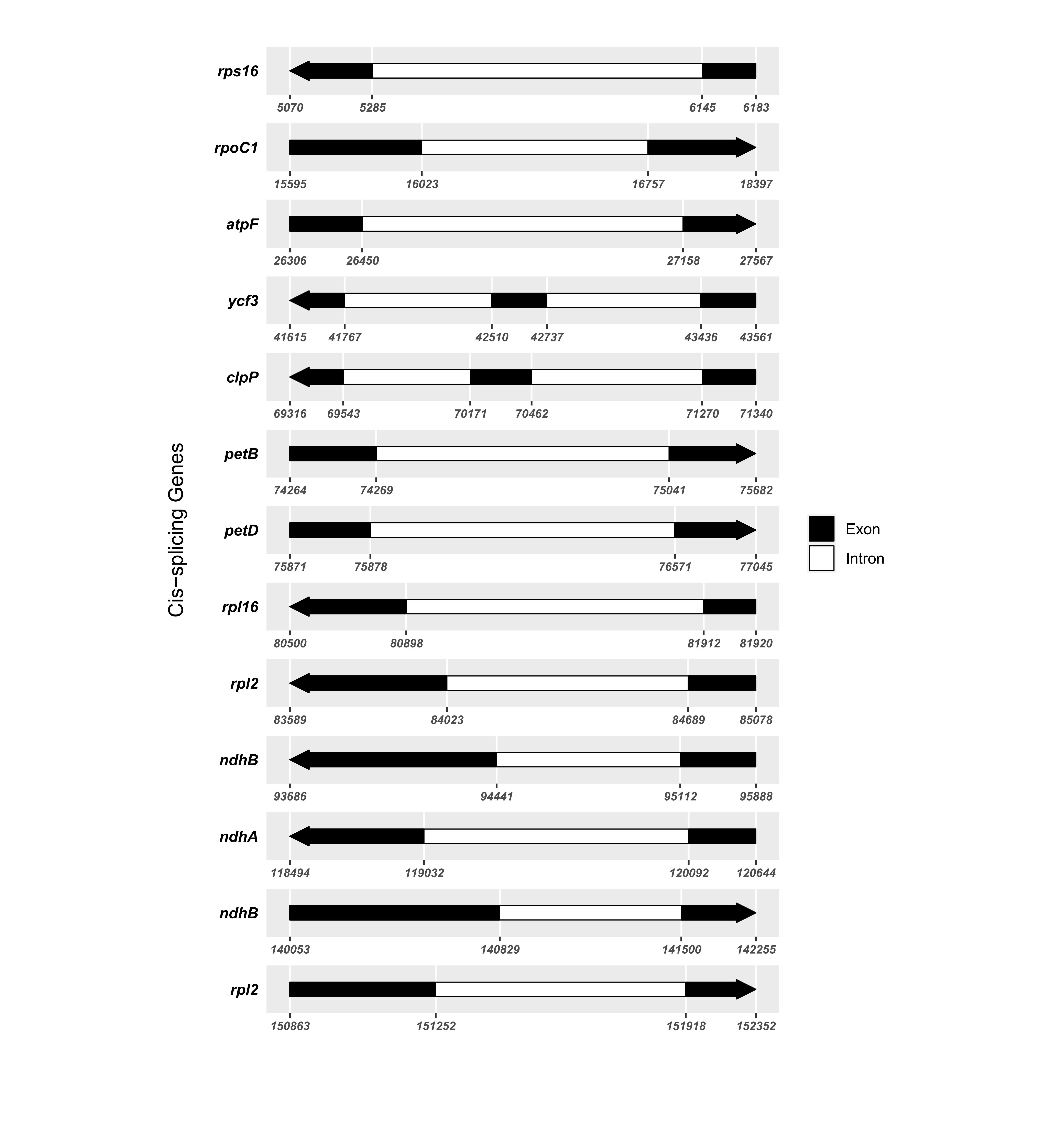


**Figure S3.** Schematic map of the cis-splicing genes in the chloroplast genome. The genes are arranged from top to bottom based on their order on the chloroplast genome.The gene names are shown on the left, and the gene structures are on the right. The exons are shown in black; the introns are shown in white. The arrow indicates the sense direction of the gene. Please note that lengths of exons and introns are not drawn to scale.


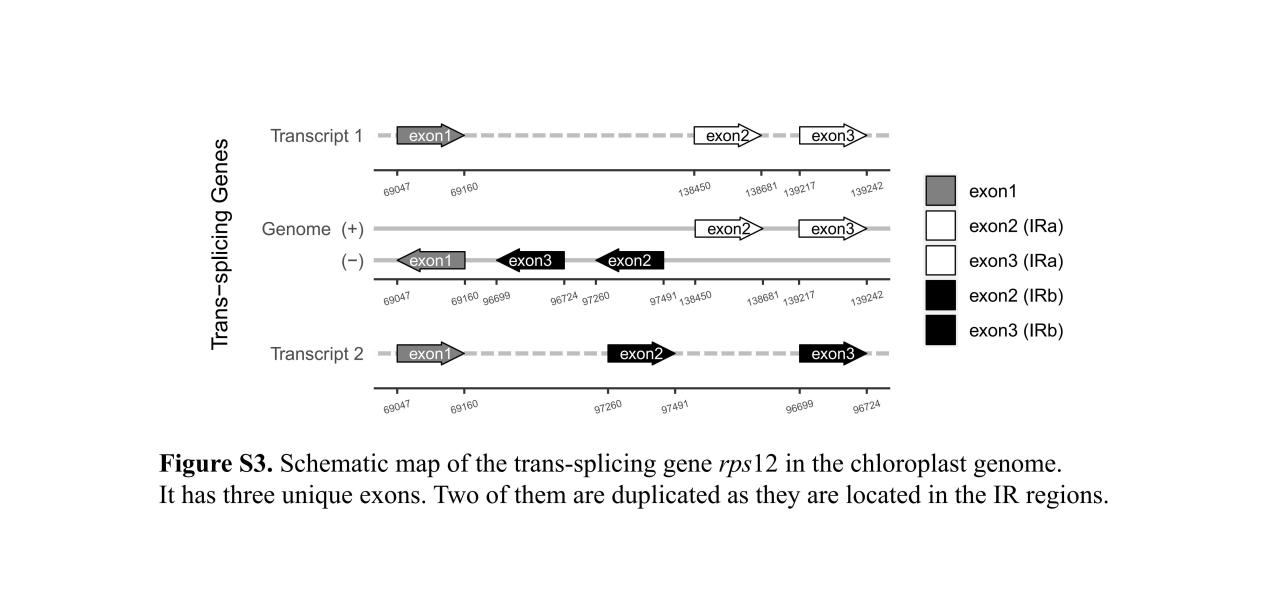


**Figure S4.** Schematic map of the trans-splicing gene rps l2 in the chloroplast genome.

It has three unique exons. Two of them are duplicated as they are located in the IR regions.

**Table S1.** 70 shared protein genes

| Type of gene | Name of gene | Number of genes |
| --- | --- | --- |
| Photosynthesis related genes | *atpA* , *atpB* , *atpE*, *atpF*, *atpH*, *atpI*, *petA*, *petB*, *petD*, *petG*, *petL*, *petN*, *psaA*, *psaB*, *psaC*, *psaI*, *psaJ*, *psbA*, *psbB*, *psbC*, *psbD*, *psbE*, *psbF*, *psbH*, *psbI*, *psbJ*, *psbK*, *psbM*, *psbN*, *psbT*, *psbZ*, *rbcL* | 31 |
| NAD(P)H dehydrogenase complex genes | *ndhA*, *ndhC*, *ndhD*, *ndhE*, *ndhF*, *ndhG*, *ndhH*, *ndhI*, *ndhJ*, *ndhK* | 10 |
| Transcription and translation related genes | *rpl14*, *rpl16*, *rpl20*, *rpl22*, *rpl2* 2, *rpl32*, *rpl33*, *rpoA*, *rpoB*, *rpoC1*, *rpoC2*, *rps11*, *rps12 2*, *rps14*, *rps15*, *rps16*, *rps18*, *rps19*, *rps2*, *rps3*, *rps4*, *rps7 2*, *rps8* | 24 |
| Other genes | *ccsA*, *cemA*, *matK*, *ycf3*, *ycf4* | 5 |
